# Pilot Randomized Controlled Study on the Effectiveness of a Virtual Reality-Based Dementia Prevention Program Using Self-Regulated Learning Strategies Among Older Adults with Mild Cognitive Impairment

**DOI:** 10.3390/healthcare13091082

**Published:** 2025-05-07

**Authors:** Ching-Hao Chang, Kuei-Yu Huang, Lou-Hui Kuo, Ya-Wen Cheng, Su-Fei Huang, Tien-Hsi Chuang, Chiu-Mieh Huang, Jong-Long Guo

**Affiliations:** 1 Department of Health Promotion and Health Education, College of Education, National Taiwan Normal University, Taipei 106, Taiwan; 81105007e@ntnu.edu.tw (C.-H.C.); louhuikuo@gmail.com (L.-H.K.); yawencheng66@gmail.com (Y.-W.C.); 81105010e@ntnu.edu.tw (T.-H.C.); 2 School of Chinese Medicine, College of Medicine, National Yang Ming Chiao Tung University, Taipei 112, Taiwan; m015512@ms.skh.org.tw; 3 Department of Chinese Medicine, Shin Kong Wu Ho Su Memorial Hospital, Taipei 111, Taiwan; 4 Department of Intelligent Technology and Long-Term Care, MacKay Junior College of Medicine, Nursing, and Management, Taipei 112, Taiwan; s331@mail.mkc.edu.tw; 5 Institute of Clinical Nursing, College of Nursing, National Yang Ming Chiao Tung University, Taipei 112, Taiwan

**Keywords:** virtual reality, mild cognitive impairment, dementia prevention, self-regulated learning, health literacy, older adults, digital health, immersive learning, randomized controlled trial

## Abstract

Background/Objectives: Dementia is a growing public health issue, especially in rapidly aging societies like Taiwan, where nearly 10% of adults over 65 show signs of cognitive decline. Given that mild cognitive impairment (MCI) serves as a critical stage for early intervention, this study examined the feasibility and preliminary effectiveness of a virtual reality (VR)-based dementia prevention program, specifically designed based on self-regulated learning (SRL) principles to enhance dementia knowledge, health literacy, and self-efficacy among older adults with MCI. Methods: A pilot randomized controlled trial (RCT) was conducted with 60 older adults aged 65 and above with MCI. Participants were randomly assigned to either an experimental group, which received a VR-based dementia prevention program, or a comparison group, which received routine paper-based educational materials. Results: The experimental group demonstrated significant improvements in overall dementia knowledge and all subdomains. Significant gains were also observed in critical health literacy and self-efficacy, though no significant changes were found in overall health literacy. Conclusions: The preliminary findings suggest that the SRL-informed VR program showed initial effectiveness in enhancing dementia knowledge, critical health literacy, and self-efficacy among older adults with MCI, highlighting its potential as an innovative approach to dementia prevention education.

## 1. Introduction

Dementia is a progressive brain disorder affecting memory, cognition, language, and behavior. According to the World Health Organization, approximately 55 million people worldwide were living with dementia in 2019, with projections estimating that this number will rise to 78 million by 2030 and 139 million by 2050 [1]. The condition poses significant challenges for public health, particularly in aging societies like Taiwan, where 10% of individuals over 65 and 30% over 80 are affected [2]. Dementia often manifests in behavioral and psychological symptoms, such as emotional instability, aggression, and communication difficulties, which contribute to caregiver stress, including feelings of anger, depression, and fatigue [3]. These issues emphasize the urgent need for effective interventions and health education strategies.

Dementia prevention and early intervention are essential strategies for reducing the disease burden. Mild cognitive impairment (MCI) represents an early transitional stage between normal aging and dementia, characterized by mild deficits in memory or other cognitive domains without significant impairment of daily functioning [4]. Approximately 10–15% of individuals with MCI progress to Alzheimer’s disease annually; however, appropriate early interventions can stabilize or even improve cognitive function in some individuals [5]. Health education has demonstrated significant benefits by enhancing individuals’ dementia-related knowledge, health literacy, and self-management skills, thereby improving quality of life and delaying disease progression [6,7]. Tailored educational strategies incorporating oral, written, and interactive methods have proven especially effective in enhancing comprehension and overcoming cognitive barriers among older adults with MCI [8]. Thus, equipping older adults with comprehensive dementia prevention education remains a critical public health priority.

Self-regulated learning (SRL) is an active and constructive process where learners establish personal learning goals and actively monitor, regulate, and control their cognitive processes, motivation, and behavior, all influenced by their goals and the surrounding environmental context [9]. Effective SRL includes structured goal-setting, systematic self-monitoring, reflective evaluation, and adaptive learning behaviors shaped by feedback and self-assessment [10,11]. Older adults tend to benefit from a balance of structured guidance and opportunities for self-regulated practice, especially when emotional support is provided [12]. Recent research suggests that virtual reality (VR) environments can effectively support SRL by increasing engagement and enabling self-paced, interactive learning, particularly where traditional instruction is limited [13]. However, much of the current SRL literature has focused on student populations or digital natives using AR or online platforms [14], leaving a gap in understanding how SRL strategies may function within immersive VR environments tailored for cognitively vulnerable older adults.

VR, as a fully immersive technology, enables users to engage with simulated environments that support experiential learning. It is increasingly recognized as a valuable tool in mental health interventions for individuals with dementia. In the field of dementia mental health, VR is regarded as an effective tool, holding potential for supporting cognitive training, improving memory and attention, enhancing mood, reducing apathy, and mitigating depressive symptoms by recreating safe, engaging, nature-based scenarios that foster emotional well-being and social interaction [15,16,17,18]. Beyond clinical applications, VR has also emerged as a promising tool in health education, providing benefits in knowledge acquisition, skill development, and learner engagement compared to traditional approaches [19].

In dementia prevention, VR has been demonstrated to stimulate cognitive processes, replicate real-life care scenarios, and alleviate behavioral and psychological symptoms [20,21]. A meta-analysis reported small-to-medium positive effects on cognition and physical fitness in individuals with MCI or dementia, particularly when using semi-immersive systems [22]. Older adults, including those with MCI, also report high acceptance of VR, citing lower discomfort and greater motivation [23]. However, most prior research has focused on global cognition or executive function, with limited attention paid to health literacy and self-efficacy—both vital for dementia prevention [20]. While VR has shown promise in clinical training and dementia care, existing research has largely focused on healthcare professionals, leaving a gap in evidence for older adults with MCI.

To address this, the present study aimed to evaluate the effectiveness of a SRL-informed VR dementia prevention program in enhancing dementia-related knowledge, health literacy, and self-efficacy among older adults with MCI in Taiwan. It was hypothesized that, compared to those receiving standard paper-based educational materials, participants in the VR intervention group would demonstrate significantly greater improvements in dementia knowledge, health literacy, and self-efficacy following the intervention.

## 2. Materials and Methods

### 2.1. Study Design

This study adopted a two-arm, open-label pilot randomized controlled trial (RCT) design to evaluate the feasibility and preliminary effectiveness of a VR-based dementia prevention program among older adults with MCI. Given the nature of the intervention, blinding of participants and facilitators was not feasible. The trial followed a parallel-group design with pre- and post-intervention assessments.

A total of 76 individuals were assessed for eligibility. Four participants were excluded, with two not meeting the inclusion criteria and two declining to participate. The remaining 72 participants were randomly assigned to either the experimental group (*n* = 34) or the comparison group (*n* = 38) using a simple coin toss method conducted by a designated research assistant who was not involved in the outcome assessment, ensuring equal probability of assignment while maintaining the simplicity needed for a small-scale feasibility trial [24]. Group assignments were not disclosed to the personnel administering post-intervention questionnaires and interviews to minimize detection bias. The experimental group received the VR-based dementia prevention program using SRL strategies, while the comparison group received traditional routine, paper-based health education materials. Trial registration: ClinicalTrials.gov NCT06888986, date: 15 March 2025. Retrospectively registered.

The estimated sample size required to achieve a statistical power of 0.9 at a significance level of 0.05 for a two-tailed test was 31 participants per group, based on an effect size (g = 0.38) derived from a meta-analysis on immersive virtual reality learning interventions [25], which served as a reference point for this study. However, considering the pilot nature of this trial, which primarily aimed to assess feasibility and preliminary intervention effects rather than establishing definitive efficacy, a smaller sample was considered acceptable. Ultimately, 60 participants were enrolled, including 30 in the experimental group and 30 in the comparison group.

### 2.2. Participants

The participants were older adults aged 65 and older, recruited from community centers, long-term care facilities, and adult day care centers in northern Taiwan. To initiate recruitment, the research team first contacted these institutions, explained the study objectives, and provided the inclusion criteria. Institutional staff then assisted in identifying potentially eligible individuals from their service populations. Trained research personnel subsequently conducted on-site screening to verify eligibility.

Prior to enrollment, all potential participants received a detailed explanation of the study’s purpose, procedures, potential risks, and benefits. Written informed consent was obtained from each participant before data collection.

The inclusion criteria were as follows: (a) being an older adult aged 65 years or older and (b) having an Eight-item Informant Interview to Differentiate Aging and Dementia (AD8) score of 2 or higher, indicating potential cognitive impairment [26]. Exclusion criteria included the following: (a) inability to use VR equipment, (b) diagnosis of severe mental disorders, (c) unconsciousness or serious physical conditions preventing participation, and (d) inability to communicate in Mandarin or Taiwanese.

### 2.3. Study Procedure

**Figure 1:**
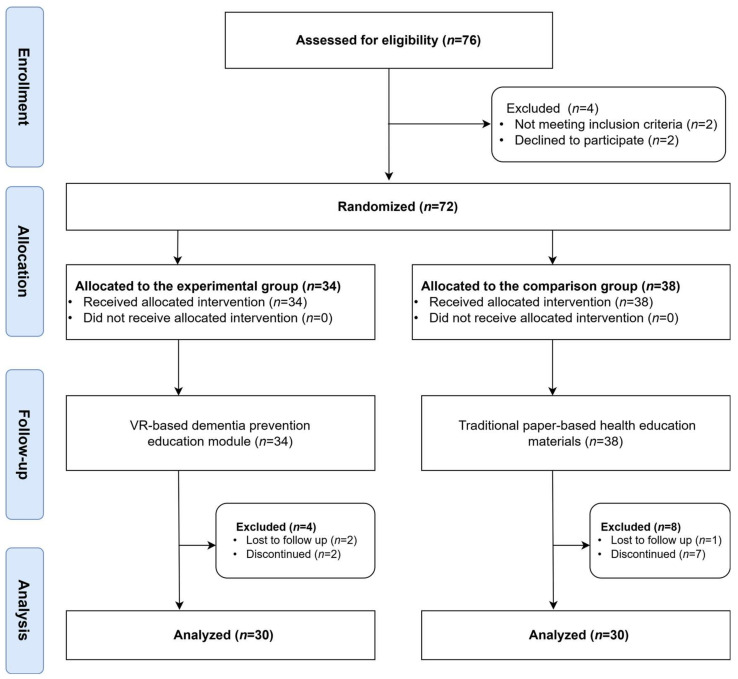
CONSORT flow diagram of participant recruitment and intervention.

Figure 1 illustrates the entire study procedure. Upon the initiation of the study, trained research personnel provided participants with a thorough explanation of the study’s objectives, procedures, and potential risks and benefits. Written informed consent was obtained prior to enrollment. Eligible participants were then randomly assigned to either the experimental group or the comparison group using the coin toss method to ensure equal allocation probability.

Before the intervention, all participants completed a pre-test assessment. The experimental group received the VR-based dementia prevention program, delivered through Meta Quest 3 VR headsets. This program included five structured units, covering dementia-related knowledge, disease management and prevention strategies, health literacy, self-efficacy, and acceptance of dementia. Participants engaged with the VR system in a controlled environment under the supervision of a facilitator, ensuring proper navigation and interaction with the learning material. The comparison group received standard, institution-provided traditional paper-based health education materials, as typically offered by their community centers, long-term care facilities, or adult day care centers.

After the intervention, all participants completed post-test assessments to evaluate changes in dementia knowledge, health literacy, and self-efficacy. The personnel conducting the assessments were blinded to group allocation to maintain data integrity and reduce bias. Any usability issues that arose during the VR sessions were documented for further analysis.

### 2.4. Intervention Development

The selection of topic and activities of the VR-based dementia prevention program, based on SRL theory, was guided by an interdisciplinary team of specialists in dementia care, health education professionals, and VR technology developers. The five structured units were developed based on evidence-based guidelines for dementia education and aligned with key domains frequently emphasized in the literature [27,28,29,30]. The program consisted of five structured units addressing critical learning objectives shown in Table 1, including insight, dementia-related knowledge, health literacy, self-efficacy enhancement, and acceptance of dementia.

Each immersive scenario was developed based on real-world cases commonly encountered by individuals with MCI or early-stage dementia, as identified and shared by clinical experts in dementia care within the research team. The scenarios were structured into narrative-based modules that reflect everyday challenges, such as managing emotional responses, coping with behavioral symptoms, and communicating needs. Each unit featured clearly defined learning goals and was integrated into a sequential learning path using scenario-based storytelling.

Delivered through the Meta Quest 3 VR headset (Meta Platforms, Inc., Menlo Park, CA, USA), the program provided an engaging, interactive learning environment. Interactive features included goal setting at the beginning of each unit, in-scenario decision points, and gamified embedded quizzes that provided immediate feedback. Visual cues and progress trackers were also incorporated to enhance self-monitoring and metacognitive regulation.

SRL strategies, including structured goal-setting, self-monitoring, embedded feedback, and guided reflective exercises, were intentionally incorporated throughout the program to enhance learning engagement and retention. The program incorporated interactive quizzes embedded throughout VR sessions to enhance self-regulation processes by supporting accurate monitoring and promoting better learning outcomes [31]. Each quiz provided immediate reinforcing feedback, including positive audio cues for correct responses and supportive explanations for incorrect responses. Additionally, key information and instructional prompts were presented as subtitles during the learning process, providing ongoing cognitive scaffolding and assisting participants in staying oriented across the various segments. This design is supported by empirical research revealing that combining timely feedback with structured prompts can improve learning performance and meta-comprehension accuracy over time [32].

Following each VR session, trained facilitators guided participants through oral reflection exercises utilizing concept mapping techniques grounded in SRL [33]. This process encouraged participants to summarize crucial points, raise relevant questions, and reflectively connect the educational content to their own experiences, promoting deeper cognitive integration and learning retention. Facilitators also tracked participants’ learning progress in real time via the Meta Horizon app and provided verbal and emotional support when needed, particularly for participants unfamiliar with VR or experiencing disorientation.

**Figure 2:**
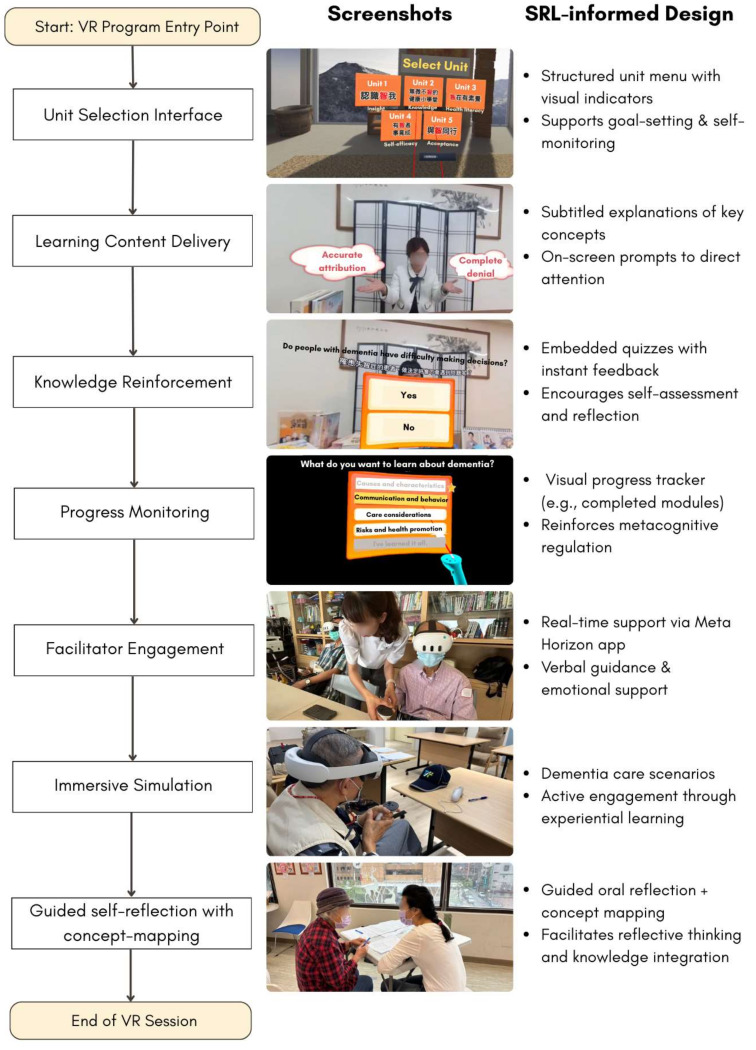
Screenshots and photos illustrating the SRL-informed design and implementation of the VR dementia prevention program.

The program underwent iterative refinement to ensure content validity and usability based on professional review and user testing. All content was reviewed by two dementia care specialists and two senior health educators. Feedback from a prior pilot user experience study informed improvements to interface design, navigation clarity, and user comfort during the VR sessions [34]. Figure 2 presents representative screenshots and real-world photos illustrating each phase of the program alongside corresponding SRL design principles, clearly demonstrating the alignment between program content, delivery structure, and targeted learning outcomes.

### 2.5. Measurements

In addition to demographic information, including age, gender, marital status, education level, past experience with dementia-related health education, relatives diagnosed with dementia, and perceived health status, several instruments were used to assess dementia knowledge, health literacy, and self-efficacy.

#### 2.5.1. Dementia-Related Knowledge

To evaluate participants’ understanding of dementia, a 25-item questionnaire was adapted from a validated instrument developed by Annear et al. (2017), and refined through expert consultation [28]. The scale assessed knowledge across four key domains: (1) causes and characteristics, (2) communication and behavior, (3) care considerations, and (4) risks and health promotion. Participants responded to each statement using a true/false format, with an additional “don’t know” option to account for uncertainty. The instrument was reviewed by three independent experts in dementia care and health education to ensure content relevance and clarity. Internal consistency reliability was excellent, with a Cronbach’s alpha coefficient of 0.98, indicating high internal coherence among items [35].

#### 2.5.2. Dementia-Related Health Literacy

Health literacy is crucial for dementia prevention, as it allows individuals to manage their health effectively [36]. We assessed health literacy using a 20-item Likert-type scale, based on Nutbeam’s framework for functional, communicative, and critical health literacy [37]. The scale was adapted from prior validated instruments and modified to reflect dementia-specific contexts [29,38]. This scale measures participants’ ability to access, understand, and apply health information related to dementia care. The scale was adapted from previous studies to ensure validity and reliability. The scale’s internal consistency was confirmed with a Cronbach’s alpha of 0.95.

#### 2.5.3. Self-Efficacy

Self-efficacy is a key factor in empowering individuals to take action and engage in dementia care practices. To measure participants’ confidence in managing dementia-related tasks, we utilized a 6-item Likert-type scale adapted from a previous validated self-efficacy instrument [39]. The items were revised to reflect the context of dementia, focusing on participants’ perceived ability to understand dementia symptoms, responding to behavioral changes, and supporting care decisions. The adapted version was reviewed by dementia care experts to ensure relevance and clarity. The Cronbach’s alpha was 0.89, confirming its reliability in this context.

### 2.6. Data Collection and Analysis

Data were analyzed using IBM SPSS Statistics 27.0. Descriptive statistics were employed to summarize demographic characteristics and baseline measures of dementia knowledge, health literacy, and self-efficacy. Independent sample *t*-tests and Chi-square tests were applied to examine group differences in demographic characteristics. Generalized estimating equations (GEE) were employed to assess intervention effects over time, incorporating a group × time interaction term to evaluate differential changes between the experimental and comparison groups. The model reported regression coefficients (β), standard errors (SE), Wald chi-square statistics, and *p*-values for each outcome variable. A significance level of *p* < 0.05 was set for all analyses. Participants with missing outcome data were excluded listwise. Baseline demographic comparisons showed no significant between-group differences; thus, no covariates were included in the model.

### 2.7. Ethical Considerations

This study was conducted in accordance with the Declaration of Helsinki and was approved by the Institutional Review Board (IRB) of National Taiwan Normal University (Approval No. 202212HM033). 

## 3. Results

### 3.1. Demographic Characteristics

The final sample included 60 older adults, comprising 30 participants in the experimental group and 30 in the comparison group. The mean age was 76.83 years (SD = 6.70) for the experimental group and 76.67 years (SD = 7.01) for the comparison group. No significant age difference was found between the groups (t = −0.94, *p* = 0.925).

In both groups, the majority of participants were female (80.00%), and gender distribution did not differ significantly between groups (χ^2^ = 0.000, *p* = 1.000). The distribution of marital status was similar across groups, with the majority being either widowed or married (χ^2^ = 2.072, *p* = 0.558). Participants were fairly evenly distributed across levels, with the largest proportion in each group reporting elementary or high school education. No statistically significant differences in educational level were found between the groups (χ^2^ = 0.328, *p* = 0.988).

Past experience with dementia-related health education was reported by 40.0% of the experimental group and 27.59% of the comparison group; however, this difference was not statistically significant (χ^2^ = 1.014, *p* = 0.314). Similarly, while a higher proportion of participants in the comparison group reported having a relative diagnosed with dementia (24.14% vs. 6.67%), this difference was not significant (χ^2^ = 4.109, *p* = 0.128).

Overall, no significant differences were found between the experimental and comparison groups in all measured sociodemographic variables, suggesting that the groups were comparable at baseline. Detailed participant characteristics are summarized in Table 2.

### 3.2. Intervention Effects on Dementia Knowledge, Health Literacy, and Self-Efficacy

Table 3 presents the Generalized Estimating Equation (GEE) analysis results, comparing outcomes between the experimental and comparison groups. For dementia-related knowledge, a significant interaction effect was observed (β = 5.333, *p* < 0.001), indicating more significant gains in the experimental group following the intervention. Subdomain analyses further revealed significant group-by-time interaction effects in causes and characteristics (β = 1.767, *p* < 0.001), communication and behavior (β = 1.167, *p* = 0.008), care considerations (β = 1.400, *p* = 0.002), and risks and health promotion (β = 1.000, *p* = 0.005), suggesting broad improvements across knowledge categories.

In terms of health literacy, although the overall score did not show a significant interaction effect (β = 6.700, *p* = 0.138), the critical health literacy subdomain showed a significant group-by-time interaction (β = 3.700, *p* = 0.032), indicating the intervention’s potential in enhancing participants’ ability to evaluate and apply health information related to dementia critically. No significant effects were observed in the functional or interactive health literacy subdomains.

For self-efficacy, the group-by-time interaction was statistically significant (β = 4.200, *p* = 0.008), demonstrating a positive effect of the VR-based program on participants’ confidence in managing dementia-related tasks and situations.

Overall, these findings suggest that the VR-based dementia prevention program effectively improved dementia knowledge across all major subdomains and significantly enhanced both critical health literacy and self-efficacy among older adults.

## 4. Discussion

This pilot randomized controlled trial evaluated the preliminary effectiveness of a VR-based dementia prevention program specifically informed by SRL principles among older adults with MCI. The baseline demographic characteristics did not differ significantly between the experimental and comparison groups, ensuring that the observed intervention effects were not due to differences in group composition. The findings suggest promising preliminary effectiveness, particularly in enhancing dementia knowledge, critical health literacy, and self-efficacy.

The intervention demonstrated significant improvements in dementia-related knowledge across all major subdomains, including causes and characteristics, communication and behavior, care considerations, and risks and health promotion. These findings support the view that immersive and scenario-based VR program—such as situations involving everyday challenges commonly faced by older adults with dementia (e.g., forgetting to turn off appliances)—can effectively strengthen emotional engagement and deepen cognitive processing in older adults. Prior studies have shown that emotionally relevant contexts enhance knowledge retention, especially in aging populations [40]. Furthermore, the integration of structured prompts and immediate feedback within the VR quizzes likely contributed to improved learning performance by guiding learners’ attention and reinforcing self-monitoring processes. Research in SRL has demonstrated that combining prompts with timely feedback can significantly enhance meta-comprehension accuracy and learning outcomes across multiple sessions, particularly by facilitating learners’ recalibration of internal monitoring and correction of misconceptions during learning [32]. These interactive features, therefore, may have played a critical role in sustaining cognitive engagement and promoting more effective knowledge acquisition in this population.

Although overall health literacy scores did not reveal a significant improvement, a notable improvement was observed in the subdomain of critical health literacy. This indicates that the VR-based intervention effectively improved participants’ ability to analyze, evaluate, and apply dementia-related health information to real-life decision-making. These gains may be attributed not only to the immersive and engaging nature of VR—shown in previous research to support comprehension and communication in health education contexts [41]—but also to the incorporation of SRL strategies within the program. Specifically, repeated self-monitoring through embedded quizzes, reflective concept mapping activities, and structured prompts likely fostered more active cognitive engagement and improved meta-cognitive awareness [31,32,33,42]. These findings echo prior research emphasizing that critical health literacy—more than self-efficacy or social support—is strongly linked to improved self-management behaviors in individuals with chronic conditions [43], highlighting the potential of immersive, self-regulated learning tools in dementia education.

A particularly encouraging result was the notable improvement in self-efficacy seen in the experimental group. This aligns with the previous literature indicating that self-efficacy is a vital predictor of sustained engagement in dementia-preventive behaviors [44]. The integration of interactive features, structured scaffolding, and guided reflective activities likely contributed to this gain by fostering learners’ confidence in applying what they learned. Recent meta-analytic evidence also confirms that SRL-based interventions can moderately enhance self-efficacy, especially when they scaffold self-monitoring and reflective evaluation [45]. Moreover, these findings align with Zimmerman’s SRL framework, which highlights the reciprocal link between self-efficacy and strategy use throughout learning cycles [46]. Additionally, this finding supports previous evidence that self-efficacy mediates the impact of health literacy on health behaviors and decision-making, particularly in older populations [47].

Similar improvements in health knowledge, health literacy, and self-efficacy have been reported in other domains of immersive VR applications. For instance, in nursing education, VR-based training has shown advantages over traditional methods in enhancing learner confidence and knowledge acquisition [48]. A recent scoping review also noted that VR interventions in chronic pain management effectively deliver health knowledge and support the development of health literacy and self-management skills, particularly through immersive and emotionally engaging experiences [49].

These findings indicate that a brief SRL-informed VR intervention can lead to significant cognitive and behavioral improvements, especially in dementia knowledge, critical health literacy, and self-efficacy. The results highlight the significance of immersive, scaffolded learning environments tailored for cognitively vulnerable older adults, adding to the growing evidence that supports SRL-based digital health education in the relatively underexplored area of dementia prevention for individuals with MCI.

Despite promising findings, several limitations should be acknowledged. As a pilot study, the relatively small and geographically limited sample restricts the generalizability of the results. The short intervention period and reliance on self-reported outcomes may also limit the conclusions regarding long-term effects. In addition, participants and facilitators were not blinded, and individuals unable to use VR equipment were excluded for feasibility reasons, which may have introduced some bias. Although the trial was registered retrospectively, the study adhered to a pre-established protocol. Future research should aim for larger, more diverse samples, include longitudinal follow-ups, incorporate objective outcome measures, and explore strategies to enhance accessibility and minimize potential selection bias.

## 5. Conclusions

This pilot study provides preliminary evidence supporting the effectiveness of a VR-based dementia prevention education program grounded in SRL theory for older adults. The SRL-informed features included in this program—such as structured goal-setting, frequent self-monitoring through interactive quizzes with immediate reinforcing or supportive feedback, and guided reflection exercises facilitated by concept mapping—collectively contributed to significant improvements in dementia-related knowledge, critical health literacy, and self-efficacy among older adults with MCI. These early insights support the potential of immersive, interactive VR technologies in dementia education, but further large-scale, longitudinal studies are needed to validate the program’s long-term impact and broader applicability.

## Figures and Tables

**Table 1:** Content of the VR-based dementia prevention program.

Unit	Learning Objective	Outcome Variable
1	▪ Recognize that disease insight describes a patient’s comprehension and acceptance of their condition, divided into six stages. ▪ Encourage a positive attitude toward dementia.	Insight
2	Acquire knowledge about dementia, encompassing its causes, characteristics, communication and behavioral aspects, care considerations, risks, and preventive methods.	Dementia-related knowledge
3	▪ Cultivate the skills to understand and utilize dementia-related information for effective decision-making (functional health literacy). ▪ Cultivate critical thinking skills and the ability to apply new information in different contexts (critical health literacy). ▪ Improve the ability to obtain dementia-related information through various communication channels (interactive health literacy).	Health literacy
4	Foster confidence and a sense of capability in accomplishing specific tasks or goals.	Self-efficacy
5	▪ Promote acceptance of dementia on an individual level. ▪ Encourage individuals to be open about sharing their condition with others and seeking the support they need.	Acceptance of dementia

**Table 2:** Demographic characteristics of participants.

Variables	Experimental Group (*n* = 30, 50%)	Comparison Group (*n* = 30, 50%)	χ^2^/t	*p*-Value
Age (years), mean (SD)	76.83 (6.70)	76.67 (7.01)	−0.094	0.925
Gender, *n* (%)			0.000	1.000
Male	6 (20.00)	6 (20.00)		
Female	24 (80.00)	24 (80.00)		
Marital status, *n* (%)			2.072	0.558
Unmarried	0 (0.00)	2 (6.67)		
Married	13 (43.33)	12 (40.00)		
Divorced or separated	1 (3.33)	1 (3.33)		
Widowed	16 (53.33)	15 (50.00)		
Educational level, *n* (%)			0.328	0.988
Elementary school	8 (27.59)	9 (32.14)		
Middle school	4 (13.79)	4 (14.29)		
High school	9 (31.03)	9 (32.14)		
University and above	8 (27.59)	6 (21.43)		
Past experience with dementia-related health education, *n* (%)			1.014	0.314
Yes	12 (40.0)	8 (27.59)		
No	18 (60.0)	21 (72.41)		
Relatives diagnosed with dementia, *n* (%)			4.109	0.128
Yes	2 (6.67)	7 (24.14)		
No	23 (76.67)	16 (55.17)		
Not sure	5 (16.67)	6 (20.69)		

**Table 3:** GEE ^a^ results for intervention effectiveness.

Variables	Coefficient (β)	SE	Wald χ^2^	*p*-Value
Dementia-related Knowledge (0–25 points)				
Group (Experimental group) ^b^	−0.400	1.229	0.106	0.745
Time (Post-test) ^c^	0.700	0.640	1.197	0.274
Group (Experimental) × Time (Post-test) ^d^	5.333	1.359	15.391	<0.001 ***
Causes and Characteristics (0–7 points)				
Group (Experimental group) ^b^	−0.333	0.428	0.606	0.436
Time (Post-test) ^c^	0.567	0.244	5.415	0.020 *
Group (Experimental) × Time (Post-test) ^d^	1.767	0.503	12.318	<0.001 ***
Communication and Behavior (0–6 points)				
Group (Experimental group) ^b^	−0.367	0.384	0.910	0.340
Time (Post-test) ^c^	−0.033	0.186	0.032	0.857
Group (Experimental) × Time (Post-test) ^d^	1.167	0.443	6.943	0.008 **
Care Consideration (0–6 points)				
Group (Experimental group) ^b^	−0.033	0.415	0.006	0.936
Time (Post-test) ^c^	−0.067	0.320	0.044	0.835
Group (Experimental) × Time (Post-test) ^d^	1.400	0.450	9.664	0.002 **
Risks and Health Promotion (0–6 points)				
Group (Experimental group) ^b^	0.333	0.390	0.730	0.393
Time (Post-test) ^c^	0.233	0.187	1.562	0.211
Group (Experimental) × Time (Post-test) ^d^	1.000	0.360	7.710	0.005 **
Health Literacy (20–100 points)				
Group (Experimental group) ^b^	0.333	4.944	0.005	0.946
Time (Post-test) ^c^	−0.867	2.398	0.131	0.718
Group (Experimental) × Time (Post-test) ^d^	6.700	4.518	2.199	0.138
Functional Health Literacy (8–40 points)				
Group (Experimental group) ^b^	0.367	1.971	0.035	0.852
Time (Post-test) ^c^	−0.533	0.947	0.317	0.573
Group (Experimental) × Time (Post-test) ^d^	0.933	1.695	0.303	0.582
Critical Health Literacy (7–35 points)				
Group (Experimental group) ^b^	0.067	1.902	0.001	0.972
Time (Post-test) ^c^	−0.600	0.953	0.396	0.529
Group (Experimental) × Time (Post-test) ^d^	3.700	1.728	4.584	0.032 *
Interactive Health Literacy (5–25 points)				
Group (Experimental group) ^b^	−0.100	1.458	0.005	0.945
Time (Post-test) ^c^	0.267	1.052	0.064	0.800
Group (Experimental) × Time (Post-test) ^d^	2.067	1.734	1.421	0.233
Self-efficacy (6–30 points)				
Group (Experimental group) ^b^	−1.267	1.633	0.602	0.438
Time (Post-test) ^c^	−0.033	0.947	0.001	0.972
Group (Experimental) × Time (Post-test) ^d^	4.200	1.588	6.997	0.008 **

Note: * *p* < 0.05; ** *p* < 0.005; *** *p* < 0.001. ^a^ GEE: generalized estimating equation; ^b^ Reference group (group): comparison group; ^c^ Reference group (time): pre-test; ^d^ Reference group (group × time): comparison group × pre-test.

## Data Availability

The data presented in this study are available from the corresponding author upon reasonable request. The data are not publicly available due to privacy restrictions.

## Abbreviations

The following abbreviations are used in this manuscript:

MCI	Mild cognitive impairment
SRL	Self-regulated learning
